# Road of Large Language Model: Source, Challenge, and Future Perspectives

**DOI:** 10.34133/research.0655

**Published:** 2025-07-29

**Authors:** Wei Zhao, Xin Yang, Zhihan Lyu, Cai Xu, Ziyu Guan

**Affiliations:** School of Computer Science and Technology, Xidian University, Xi’an, China.

## Abstract

Language model (LM), a foundational algorithm in the development of capable artificial intelligence, has been widely explored, achieving remarkable attainment. As research advances, large language models (LLMs) have emerged by pretraining transformer-based models on large-scale corpora. These models showed great zero-shot and few-shot learning capabilities across a variety of tasks, attracting widespread attention from both academia and industry. Despite impressive performance, LLMs still tackle challenges in addressing complex real-world scenarios. Recently, the advent of DeepSeek has reignited intense interest among researchers. In this paper, we provide a concise development history of LLM and discuss current challenges and future perspective. In practice, we focus on 4 crucial aspects of LLMs, including emergent abilities, human alignment, retrieval augmented generation, and applications in specific domains.

## Introduction

**Fig. 1.**
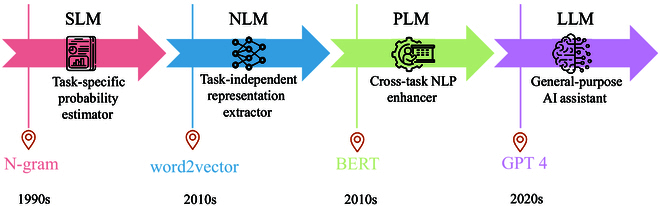
The evolutionary process of LM. Notably, the time periods assigned to each stage may not be entirely precise. The timeframe is primarily determined based on the publication dates of the representative studies at each stage, serving as a general reference rather than an exact chronological delineation.

Generally, LMs primarily aim to predict the generative likelihood of subsequent tokens. In early attempt, statistical language models (SLMs) [1] were proposed to predict the future token based on the Markov assumption. Because of SLMs, the performance of many natural language processing (NLP) tasks was improved. However, they suffered from serious exponential problem as sequence lengths increase. Hence, neural language models (NLMs) [2] were developed, which use neural networks to aggregate contextual information and to predict the likelihood of future words. Despite this advancement, due to fixed word representations, NLMs were incompetent in the issue of polysemy, where the same word exhibits different meanings in different contexts. In response, pretrained language models (PLMs) [3] were introduced, where PLM is first pretrained on unlabeled data with predefined tasks and then fine-tuned to adapt to different downstream tasks. Through scaling PLM, in terms of both model size and amount of training data, researchers found that larger PLM exhibits distinct behaviors compared to smaller ones and achieves amazing performance, leading to the development of large language models (LLMs) [4]. This progression reflects the ability of LMs to comprehend more complex content and solve general problems (see Fig. 1).

**Fig. 2.**
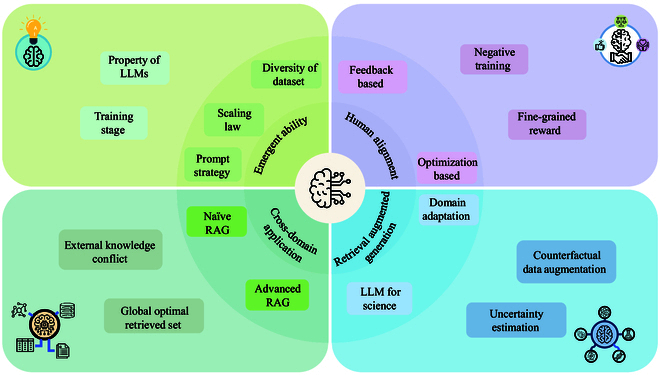
Key directions of LLMs in this paper: emergent abilities, human alignment, retrieval augmented generation, and cross-domain application. Each area highlights critical challenges and methodologies, providing a structured perspective on LLM advancements.

Nowadays, LLMs are increasingly influencing the development of artificial intelligence (AI) research, and the emergence of DeepSeek-R1 [5] and DeepSeek-V2 [6] has prompted a revolution in the popular paradigm of LLMs. Faced with both opportunities and challenges, sustained efforts in LLM research and development are crucial. This paper introduces 4 interrelated but relatively independent themes: emergence abilities, human alignment, retrieval augmented generation (RAG), and cross-domain applications, providing readers with a comprehensive view. The reason of choosing the above 4 themes can be explained by analogy with human cognition. Emergent abilities and human alignment can be considered as fundamental human capacities for understanding and communication, which are the core capabilities of artificial general intelligence (AGI). On the other hand, RAG approaches mirror how humans use external tools and knowledge to expand their own abilities. Specifically, RAG enables LLMs to solve problems, which they cannot handle based on their intrinsic abilities, and is an advanced technology. Furthermore, cross-domain applications are similar to humans applying their existing knowledge and reasoning abilities to tackle unseen challenges, which is one of the ultimate goals in the development of AI. As shown in Fig. 2 Our contributions are summarized as follows:

• We conduct a comprehensive review of contemporary developments of LLMs, with a focus on emergent abilities, human alignment, RAG, and cross-domain applications. By synthesizing a wide range of literature, we illuminate the theoretical foundation of these approaches and introduce their development history and latest progress.
• By critically assessing the inherent challenges and potential avenues for innovation, we also offer a rigorous discussion on future research directions. This exploration not only identifies unresolved challenges of existing methods but also outlines forward-looking directions that could guide subsequent advancements in investigating and utilizing LLMs.

## Exploration of Emergent Abilities: The Source of LLMs

**Fig. 3.**
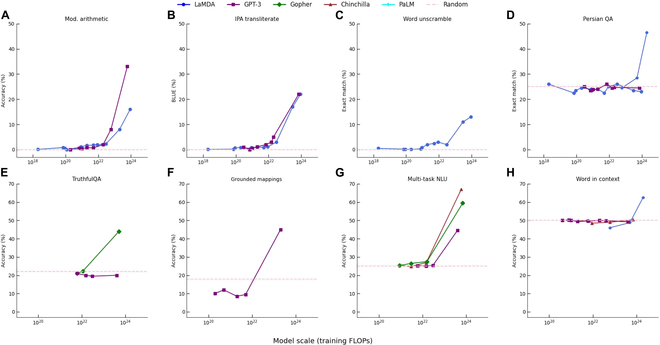
Some examples of emergent abilities in the few-shot setting (from literature [7]). LLMs exhibit emergent abilities, characterized by sharp and unpredictable performance increases on specific tasks as model scale expands. Different model families demonstrate sudden improvements in accuracy, suggesting non-linear capability emergence with increasing training FLOPs. This phenomenon is demonstrated across eight distinct benchmarks: (A) modular arithmetic, (B) IPA transliteration, (C) word unscrambling, (D) Persian QA, (E) TruthfulQA, (F) grounded mappings, (G) multi-task NLU, and (H) word in context.

In the literature [7], emergent abilities in LLMs are formally defined as “abilities that are absent in smaller models but arise in larger models”, a distinction from earlier PLMs. The ability manifests as a sudden qualitative improvement at a certain model scale and is unpredictable. As shown in Fig. 3, when the scale of the model exceeds a specific threshold, the performance of the model greatly increases. The threshold is hardly predictable and varies with different models and tasks. Based on emergent abilities, 3 prompting strategies, including in-context learning [8], instruction following [9], and chain-of-thought (COT) [10], have become widespread way to use LLMs for various downstream tasks [11–13]. However, the underlying factors contributing to emergent abilities remain unexplored. It is crucial to understand how LLMs work and how the prompt format should be structured to maximize their potential.

### Scaling law

Substantial efforts have been made to investigate the source of emergent abilities. Scaling law [14,15] is a great representative of these works, which reveals that scaling can greatly improve model capacity and eventually lead to emergent abilities. Kaplan et al. [14] firstly explore the relationship between the performsnce of LLMs and important factors. By fitting the model performance with varied data sizes (from 22 million to 23 billion tokens), model sizes (from 768 million to 1.5 billion non-embedding parameters), and training compute, they found that the model performance has a strong dependence relation on model sizes, data sizes, and training compute, which can be formulated as:

$$\begin{array}{ll} L\left(N\right) & ={\left(\frac{N_{c}}{N}\right)}^{\alpha_{N}},\;\alpha_{N}∼0.076,N_{c}∼8.8\times 10^{13}\\ L\left(D\right) & ={\left(\frac{D_{c}}{D}\right)}^{\alpha_{D}},\;\alpha_{D}∼0.095,D_{c}∼5.4\times 10^{13}\\ L\left(C\right) & ={\left(\frac{C_{c}}{C}\right)}^{\alpha_{C}},\;\alpha_{C}∼0.050,C_{c}∼3.1\times 10^{8} \end{array}$$ (1)

Later, Hoffmann et al. [15] proposed an alternative form of scaling law. Differently, they conducted rigorous experiments by varying a larger range of model sizes (from 70 M to 16 B) and data sizes (from 5 B to 500 B tokens), and fitted a similar scaling law yet with different coefficients:

$$N_{\mathrm{opt}}\left(C\right)=G{\left(\frac{C}{6}\right)}^{a},\;D_{\mathrm{opt}}\left(C\right)=G^{-1}{\left(\frac{C}{6}\right)}^{b}$$ (2)

The experiment showcased that model size makes more contribution to emergence abilities than does data size. In summary, scaling law reveals that as the number of parameters and data size increase, the model can represent more abstract language features, thereby achieving generalization to new tasks and obtaining emergence ability.

### Other factors

In addition, a myriad of factors during the training and application of LLMs have an impact on emergent capabilities. For example, the diversity and quality of training data have been shown to significantly influence emergent abilities [16]. Diverse data cover various patterns, domains, and contexts, which enable LLMs to learn general representations and provide the foundations for emergent abilities. High-quality datasets offer rich task-related context, which allows LLMs to learn different task patterns, enhances the generalization of LLMs on unseen tasks, and facilitates the emergent of LLMs. In addition to training dataset, diverse pretraining tasks are able to improve generalization of LLMs and foster the emergence of emergent abilities [7]. Specifically, instruction tuning provides comprehensive task descriptions for LLMs by designing and collecting instruction data covering multiple tasks. During the optimization phase, LLMs obtain accurate understanding of tasks, enabling LLMs to comprehend a wide range of tasks and exhibit emergent capabilities. In downstream tasks, different prompt formats, which are generated through manual [17] or automated optimization [18], tend to have different impacts on emergent abilities. As research advances, Schaeffer et al. [19] argued that LLMs do not have the so-called emergence ability and the phenomena of emergent abilities are just an illusion in the process of evaluating the performance of LLMs. By using a range of metric to evaluate the capabilities of LLMs, they found that the emergent abilities of LLMs are observed under the condition of using nonlinear or discrete metrics. In contrast, when using linear or continuous metrics, the curve of LLMs’ performance becomes smooth, continuous, and predictable. Consequently, the phenomena of emergent abilities are due to the selection of metrics rather than fundamental changes in model behavior with scaling model size and data size. Moreover, they conduct meta-analysis on open-sourced benchmarks to further prove their hypotheses. Finally, the authors extended their experiments to visual tasks, including image reconstruction and image classification. The findings suggest that emergent abilities are not unique to LLMs but rather a widespread issue inherent to evaluation methodologies.

## Understanding Human Alignment: The Soul of LLMs

Emergent abilities mark a significant milestone in the development of LLMs. However, as LLMs become increasingly powerful and autonomous, their objectives may not be consistent with human expectations. In practice, LLMs suffer from problems of undesired behaviors, biases, or even safety risks. To address these issues, human alignment research focuses on methods that guide models toward producing more reliable, ethical, and helpful outputs.

### Feedback based

**Fig. 4.**
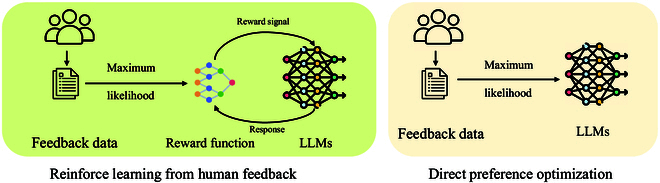
Illustrations of RLHF and DPO framework. RLHF refines LLM behavior using a reward model trained on human feedback, iteratively optimizing responses based on reward signals. In contrast, DPO simplifies the alignment process by directly optimizing model likelihood based on human preference data, eliminating the need for an explicit reward function.

Based on reinforcement learning, reinforcement learning from human feedback (RLHF) [20] regards human feedback as a reward signal to guide LLMs in generating human-aligned responses. Once proposed, RLHF quickly becomes a popular framework of human alignment (Fig. 4). However, RLHF depends heavily on high-quality human feedback, which is both costly and time-consuming due to the need for qualified annotators. To mitigate this issue, reinforcement learning from AI feedback (RLAIF) [21] was introduced. In contrast to RLHF, RLAIF employs AI agents to generate response–preference pairs rather than human annotators, which reduces the demand for human annotators and enables large-scale feedback generation.

### Optimization based

In addition to designing feedback strategies, researchers have paid attention to the impact of reinforcement learning techniques on human alignment. Given that RLHF is a complex and often unstable process, Rafailov et al. [22] proposed the direct preference optimization (DPO) algorithm, taking advantage of the mapping relationship between the reward function and the optimal policy. It demonstrates that this constrained reward maximization problem is essentially a classification problem solved on human preference data and can be precisely optimized through single-stage policy training. To improve the sampling efficiency of proximal policy optimization (PPO), Liang et al. [23] designed PTR-PPO, which combines both on-policy and off-policy methods. In practice, by introducing prioritized trajectory replay, PTR-PPO effectively reuses past experiences and improves sampling efficiency. Different from traditional RLHF approaches, which is computationally expensive, SuperHF [24] introduces a supervised fine-tuning paradigm, leading to a more data-efficient and computationally feasible human alignment. Existing human preference alignment methods assume that the preference relationships between different choices are monotonic and transitive, providing a reasonable approximation of human preferences. However, human decision-making is effected by various factors, leading to inconsistency and nonlinearity. To address this, Wu et al. [25] introduced the self-play framework SPPO. In this framework, LLMs are fine-tuned based on their own previous rounds, optimizing through model-generated synthetic data and annotations from the preference model.

## Analyzing RAG: The Extension of LLMs

**Fig. 5.**
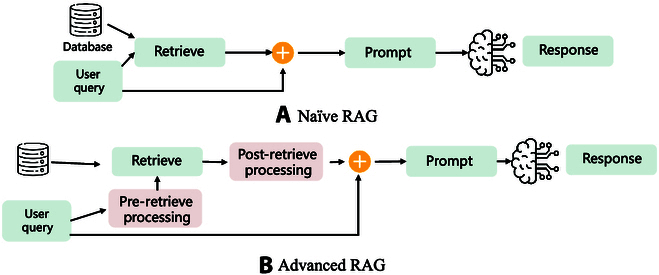
The architecture of naïve RAG and advanced RAG. (A) Naïve RAG directly retrieves information from a database and incorporates it into the prompt without additional processing. (B) Advanced RAG introduces pre-retrieval and post-retrieval processing steps to refine the retrieved content, improving relevance, coherence, and response quality. These enhancements help LLMs generate more accurate and contextually appropriate outputs.

Human alignment helps shape LLMs to better reflect human values and preferences, and mitigate the problem of unexpected output. During inference, traditional LLMs generate responses only based on their inherent knowledge, which often suffer from hallucinations and outdated information. To address these limitations, RAG equips LLMs with an external retrieval mechanism, allowing LLMs to fetch relevant information from structured knowledge graphs, web sources, or proprietary knowledge bases before generating responses (Fig. 5). Through external tools and knowledge, RAG effectively improves factual accuracy, enhances interpretability of LLMs, and reduces misinformation of output, making LLMs more reliable and adaptable to real-world applications.

### Naive RAG

In early work, the “retrieval-then-read” framework [26] was widely adopted. In this framework, a retriever is first employed to compute the similarity between the documents and the user input. The top-*k* document set with the highest similarity is then selected as the retrieval result. Finally, both the aforementioned retrieval result and user input are concatenated and fed into the LLMs to generate the response. This approach has achieved significant performance improvements in knowledge-intensive tasks. However, naive RAG encounters several notable issues, including influence of retriever’s capacity, inconsistencies between the LLM’s response and the retrieved documents, and over-reliance on the retrieval result [27]. Numerous efforts have been made to overcome the above downside.

### Advanced RAG

In “retrieval-then-read” framework, the user input is directly used to retrieve relevant documents. However, due to the complexity and ambiguity of language, such as professional terms and ambiguous abbreviations, user input may not always be suitable to serve as the query. Ma et al. [28] proposed the “Rewrite-Retrieve-Read” paradigm, which utilizes LLMs or PLMs to rewrite user inputs, making the rewritten queries more suitable for retrieval tasks, thereby improving the quality of the retrieval results. In practice, redundant information always overwhelms critical information, thereby interfering with the final response of LLM. Moreover, long contexts may lead to the “middle loss” problem [29], where LLMs always focus on the beginning and the end of texts, neglecting the middle sections. Therefore, the retrieved documents need to be further processed to help LLMs better leverage external knowledge. Jiang et al. [30] employed an additional LM to detect and remove unimportant portion. Through compression, the original prompt becomes well understood by LLMs. In the standard RAG framework, the retrieval step is usually performed only once, which only provides limited information [31]. Asai et al. [32] improve the original RAG framework by enabling LLMs to actively determine when and what should be retrieved, thus becoming sufficient for complex multi-step reasoning tasks.

## Delving into Cross-Domains Application: The Scalability of LLMs

The development in human alignment and RAG has significantly enhanced LLMs, making them aligned with human values and grounded in factual knowledge. Benefiting from broadening the applicability of LLMs in complex and knowledge-intensive fields, cross-domain application receives increasing attention from both academia and industry. By applying LLMs across different domains, they can inspire their potential, bridge knowledge gaps, and integrate expertise from multiple disciplines. Moreover, for LLMs, the cross-domain applications demonstrate that LLMs shift toward general intelligent systems, which needs numerous efforts in adaptability, reliability, safety, and capability.

### Domain adaptation

**Fig. 6.**
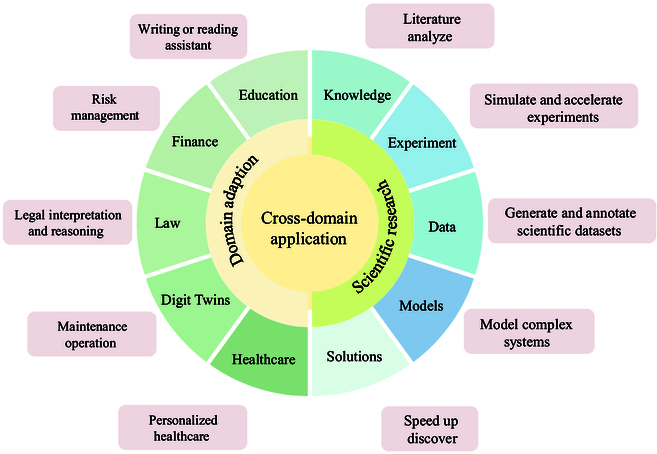
Some examples of LLMs for cross-domain application. In scientific research, LLMs contribute to literature analysis, experiment simulation, data annotation, complex system modeling, and accelerating discoveries. In domain adaptation, they enhance education, finance, law, healthcare, and risk management through tasks such as legal reasoning, personalized healthcare, and writing assistance. These applications demonstrate the versatility of LLMs in integrating and applying knowledge across diverse disciplines.

Because of the unique advantage in zero-shot and few-shot learning, LLMs are able to quickly learn and adapt to novel tasks through prompt strategy, although they have never been explicitly trained on these tasks before (Fig. 6). A number of studies have applied LLMs to the medical domain. With the development of society, mental health has attracted increasing attention. Yang et al. [33] utilize LLMs in the mental health analysis task to detect stress, depression, and suicide, achieving impressive performance. Due to the existence of specialized terminology in radiology reports, these reports are often difficult for patients without the medical background to understand. Jeblick et al. [34] employed LLMs to transform the original radiology reports into a version that is easy for patients to understand, allowing them to play a more active role in their own treatment process. Another important application domain is Digital Twins, where LLMs can analyze simulation data from Digital Twins and make fast and intelligent decisions. In smart manufacturing, for example, LLMs detect hazardous signals based on sensor data from Digital Twins, notifying administrator in time. Moreover, LLMs can be employed to perform predictive maintenance and automatically generate fault reports [35], including analysis of fault causes and maintenance history, and predict follow-up maintenance recommendations. In short, combining LLMs with Digital Twins is able to improve the degree of automation.

### LLMs for science

Interestingly, the 2024 Nobel Prizes in Physics and Chemistry were awarded to AI researchers, which sufficiently show the tremendous potential of AI for science. Because of their impressive capacity, LLMs revolutionized the way how we explore AI for scientific research. To make new discoveries, researchers have to master a preexisting knowledge system that is rapidly growing and increasingly specialized. Such “burden of knowledge” seriously hinders the development of science, since researchers spend too much on reading related literature to keep up with the latest advancements in their fields. Nowadays, researchers turn to LLM-based scientific assistants to help address the above problems. In practice, LLMs are adopted to analyze, extract, and integrate meaningful information from a mount of literature, so as to greatly enhance researchers’ work efficiency. In addition to great analytical and comprehension abilities, LLMs excel at complex reasoning tasks, allowing to accelerate the experimental process. For instance, in drug development, protein structures exhibit regularity, enabling researchers to decompose the prediction process into multiple reasoning steps and design optimal COT strategies for LLMs, thereby speeding up drug molecule discovery [36,37]. By ingesting larger volumes of collected data, LLMs can learn more powerful patterns and regularities within the data, resulting in accurately modeling these complex systems. In environmental and climate science, LLMs can be used for climate modeling and prediction, facilitating researchers to better understand the complex mechanisms of climate change and conduct weather forecasts.

## Discussion and Perspective

In this paper, we have reviewed the recent progress of LLMs in 4 aspects, including emergent abilities, human alignment, RAG, and cross-domain application. Then, we outline the discussions of this paper, introducing the challenges and future perspective for LLMs, in the following aspects.

### Overlooked factors in the study of emergent abilities

With the continuous exploration of LLMs, their properties are also constantly being revealed [38,39]. Based on these properties, we can conduct a more in-depth analysis of the emergence abilities of LLMs. For example, some works showcase that different parameters in LLMs contribute differently to outcomes [38]. Thus, we can analyze the importance of different parameters during the training stage and investigate the dynamic changes of super weight. The aforementioned experiments allow us to explore the contribution of parameter change to the emergent abilities of LLMs. Moreover, because the knowledge in LLMs is stored in feed-forward network (FFN) modules of each layer [39], the probe can be adopted to monitor FFN modules across different layers during the training phase. Therefore, we can analyze the relationship between the knowledge contained in the LLM and the emergent abilities of LLMs in the dimension of training time. In short, taking the training phase into consideration can provide interesting perspective for figuring out the source of emergent abilities.

### Advancing human alignment in LLMs

For RLHF, the feedback data for human or AI are crucial. However, in some complex scenarios, even humans struggle to effectively judge whether the outputs of LLMs align with human preferences, let alone LLMs themselves. Hence, ensuring that LLMs align with human preferences in complex scenarios is a challenge that remained to be addressed. Given the difficulty of constructing positive evaluation samples, we should shift our focus toward exploring negative samples. Current research tends to treat positive and negative evaluation samples equally. However, negative evaluation samples are generally easier to obtain compared to positive ones. Therefore, using methods such as unlearning [40] or contrastive learning [41], we can guide LLMs to avoid generating negative evaluation outputs, thereby achieving human preference alignment in complex scenarios. Discrimination of human alignment involves multiple aspects. For example, the responses generated by LLMs may conform to human values in one dimension (e.g., provide useful information) but have problems in another dimension (e.g., may introduce bias or be harmful). The reward signals from human annotators or LLMs are often a single scalar, failing to fully represent human preferences. This limitation might lead LLMs to be optimized for wrong objectives, potentially resulting in misaligned outputs. Sophisticated signals or training strategies should be designed to capture fine-grained human alignment criteria.

### Rethinking retrieval-augmented generation

The external knowledge of most existing works is based on authoritative sources such as Wikipedia. In practice, documents are from a wide range of individual users or self-media organizations, and not verified by professionals or institutions. Therefore, these retrieved documents may contain conflicting information, which hinders LLMs from integrating information from different retrieved documents. Explicitly modeling knowledge conflicts between documents is beneficial for enabling LLMs to better understand and analyze the knowledge contained in retrieved documents, thereby improving the accuracy of generated content.

Current retrieval methods, which select documents from a database based on similarity, is able to obtain documents most relevant to the query. However, the above methods just focus on the gain from an individual retrieved document, resulting in retrieving a collection of documents that are highly similar to one another. Thus, retrieval result is a locally optimal set of documents and fails to offer sufficient external knowledge to the LLMs. To address this issue, the retrieval process should consider the integral benefit of retrieved documents. While utilizing similarity as selection criterion, the knowledge contribution of each candidate document to the retrieved documents should also be evaluated so as to obtain the globally optimal set of retrieved documents.

### Enhancing the cross-domain applications of LLMs

The cross-domain application of LLMs has achieved remarkable performance. However, in some domains with small data scale, without sufficient data for fine-tuning, LLMs always fail to adequately learn new features and patterns, leading to poor performance. The above problems pose a significant challenge for generalization of LLMs. As one of the promising solutions, counterfactual data augmentation scales dataset with “counterfactual” instance. Based on hypothetical scenarios, counterfactual data augmentation alters conditions or features of input, leading to “counterfactual” samples and enriching dataset. In addition to only scaling data size, counterfactual samples introduce a wide variety of counterfactual perturbations and urge LLMs to explore causal relationships between variables within the data, reducing the LLMs’ dependence on spurious correlation that causes poor generalization. Overall, by facilitating LLMs to learn richer, more robust features and patterns, counterfactual data augmentation improves their abilities of generalization and causal reasoning. In the future, counterfactual data augmentation will be one of the key techniques for cross-domain application.

Since LLMs are essentially black box models, they suffer from the lack of explainability and transparency, and tend to be overconfident. Furthermore, since LLMs are trained on large-scale corpora, which consists of both high-quality and low-quality data, LLMs are likely to generate biased or hallucinated responses. Such limitation significantly impacts the application of LLMs in scientific community. To solve these problems, it is particularly important to conduct in-depth research on the uncertainty estimation for LLMs. Uncertainty estimation approach provides users with confidence information about the predicted results, allowing them to judge the reliability of the model output and avoid misleading conclusions. Besides, in simulation experiments, uncertainty estimation is able to indicate which prediction results need further validation or manual intervention, thereby improving the efficiency of human–machine collaboration. For LLMs, uncertainty estimation is a promising approach for improving reliability and trustworthiness, which attracts increasing attention from researchers.

### Insights from DeepSeek: Findings and implications

In recent years, substantial advancements have been achieved in the field of LLMs. Among the prominent developments, DeepSeek-R1 and DeepSeek-V2 emerge as the pioneers that investigate innovative methodologies to promote the evolution of LLMs. Through algorithmic innovation, DeepSeek demonstrates extraordinary performance and showcases its potential to extend the boundaries of natural language understanding and problem-solving.

Due to the huge number of parameters, the requirement of substantial memory footprint during inference hinders deployment of LLMs in real-world applications. Since the proposal of the LLM, boosting inference efficiency has attracted considerable attention. However, these methods often suffer from the problem of degradation, failing to achieve the desired balance between performance and efficiency. To this end, DeepSeek-V2 designs an innovative module, named multi-head latent attention (MLA) [6]. Through mapping the key-value (KV) cache into a latent vector, MLA effectively compresses the computational cost and captures long-distance dependencies. Moreover, DeepSeek-V2 also adopts the DeepSeekMoE [42] architecture, which consists of multiple expert subnetworks. The benefits of multiple expert subnetworks can be 3-fold. First, based on different inputs during inference, the model can dynamically select and activate a small subset of these experts, reducing computational cost. Meanwhile, the overall capacity of the model is also preserved. Second, different experts are assigned to capture diverse linguistic patterns, enabling flexible scaling and task-specific fine-tuning. Finally, by analyzing the activation patterns of each expert subnetwork, researchers can trace the logical pathways behind the decision-making process. Both MLA and DeepSeekMoE show the great potential of architectural optimization and sophisticated mechanisms in driving the advancement of LLMs. As algorithms become more important in the research of LLMs, we should increasingly focus on algorithmic refinement, resource economy, and interpretability measures. This shift is expected to establish the foundation for AI systems that are more accessible, efficient, and controllable, contributing to the sustainable development of robust AI.

Another interesting finding is about distillation. In DeepSeek-R1, the authors fine-tuned smaller LLMs using the data generated by DeepSeek-R1. The experimental results show that fine-tuned LLMs exhibit great reasoning abilities, which are comparable to those of large-scale LLMs. Notably, in specific tasks such as mathematical reasoning and programming, the fine-tuned smaller models even surpass many traditional open-source models. Those findings show that distillation is a promising way to improve the reasoning capabilities of smaller LLMs. Developing a DeepSeek-R1-level model from scratch can be prohibitively expensive, potentially costing millions of dollars, which may discourage many researchers with limited budgets. Distillation offers a more cost-effective option. By designing effective distillation strategies, the reasoning patterns and complex reasoning capacities of larger LLMs are transferred to smaller ones, which is more affordable. In addition to improving the reasoning performance of smaller models, distillation also greatly decreases computational costs during deployment and provides a viable path for deploying efficient reasoning models in resource-constrained environments.

### Ethical and legal risks of LLMs

Due to the impressive intelligence capacity, including complex reasoning and understanding, LLMs have attracted public attention. However, the essence of LLMs still remains to be answered. Given our limited understanding of LLMs, people often rely on some simplification approaches such as “labeling” to discuss them. Based on labeling, people use beautiful vocabulary to describe LLMs in the hope that they will eventually become “intelligent individuals”. However, the beautiful “metaphors” make people overlook the ethical and legal risks behind LLMs. On the other hand, some critics hold different opinions, regarding LLMs as systems merely performing “data fitting”. Labeling enables people to quickly understand complex systems, but it is also a double-edged sword, potentially obscuring the ethical and legal risks inherent in LLMs. If we consider LLMs as tools, then the creators of LLMs are engaging in commercial activities. The unauthorized use of public data for commercial activities in the training of LLMs has infringed upon the rights of numerous individuals or institutions. However, if LLMs are regarded as the true “individual”, their training process can be considered as “learning”, just like human, and learning on public data does not require additional payment. Moreover, personifying may blur the boundaries of responsibility between model developers and users, complicating the assignment of responsibility. When a vehicle equipped with an intelligent driving system is involved in a car accident, the owner of the vehicle may blame the intelligent driving system’s “wrong decision-making”, while the developer may claim that the system operates based on training data and predefined rules, and that the accident has more to do with the user’s usage or the specific environment. While embracing the potential of technology, we also need to maintain critical thinking to ensure that technological development always centers around serving human well-being.
